# Biomarkers of legume intake in human intervention and observational studies: a systematic review

**DOI:** 10.1186/s12263-018-0614-6

**Published:** 2018-09-10

**Authors:** Pedapati S. C. Sri Harsha, Roshaida Abdul Wahab, Mar Garcia Aloy, Francisco Madrid-Gambin, Sheila Estruel-Amades, Bernhard Watzl, Cristina Andrés-Lacueva, Lorraine Brennan

**Affiliations:** 1 UCD School of Agriculture and Food Science, UCD Institute of Food and Health, UCD, Belfield, Dublin 4, Ireland; 2 Biomarkers and Nutrimetabolomic Laboratory, Department of Nutrition, Food Sciences and Gastronomy, XaRTA, INSA, Faculty of Pharmacy and Food Sciences, University of Barcelona, Barcelona, Spain; 3 CIBER de Fragilidad y Envejecimiento Saludable (CIBERFES), Instituto de Salud Carlos III, Barcelona, Spain; 4 Department of Physiology and Biochemistry of Nutrition, Max Rubner-Institut, Federal Research Institute of Nutrition and Food, Karlsruhe, Germany

**Keywords:** Legumes, Metabolomics, Biomarkers

## Abstract

There is a growing interest in assessing dietary intake more accurately across different population groups, and biomarkers have emerged as a complementary tool to replace traditional dietary assessment methods. The purpose of this study was to conduct a systematic review of the literature available and evaluate the applicability and validity of biomarkers of legume intake reported across various observational and intervention studies. A systematic search in PubMed, Scopus, and ISI Web of Knowledge identified 44 studies which met the inclusion criteria for the review. Results from observational studies focused on soy or soy-based foods and demonstrated positive correlations between soy intake and urinary, plasma or serum isoflavonoid levels in different population groups. Similarly, intervention studies demonstrated increased genistein and daidzein levels in urine and plasma following soy intake. Both genistein and daidzein exhibited dose-response relationships. Other isoflavonoid levels such as *O*-desmethylangolensin (*O*-DMA) and equol were also reported to increase following soy consumption. Using a developed scoring system, genistein and daidzein can be considered as promising candidate markers for soy consumption. Furthermore, genistein and daidzein also served as good estimates of soy intake as evidenced from long-term exposure studies marking their status as validated biomarkers. On the contrary, only few studies indicated proposed biomarkers for pulses intake, with pipecolic acid and *S*-methylcysteine reported as markers reflecting dry bean consumption, unsaturated aliphatic, hydroxyl-dicarboxylic acid related to green beans intake and trigonelline reported as marker of peas consumption. However, data regarding criteria such as specificity, dose-response and time-response relationship, reliability, and feasibility to evaluate the validity of these markers is lacking. In conclusion, despite many studies suggesting proposed biomarkers for soy, there is a lack of information on markers of other different subtypes of legumes. Further discovery and validation studies are needed in order to identify reliable biomarkers of legume intake.

**Electronic supplementary material:**

The online version of this article (10.1186/s12263-018-0614-6) contains supplementary material, which is available to authorized users.

## Background

Legumes are fruits or seeds of a plant belonging to the family Fabaceae and are a popular food source in the traditional diets of many regions in the world. Well-known legumes include peas, beans, lentils, lupins, chickpeas, carob, soybeans, peanuts, and tamarind. They provide proteins, complex carbohydrates, and soluble and insoluble fibers. Legumes also contain a number of phytochemicals and antioxidants which include isoflavones, lignans, phytoestrogens, alkaloids, saponins, phytates, protease, and chymotrypsin inhibitors as well as micronutrients such as iron, copper, and manganese. Consumption of legumes in general plays a role in the prevention of cancer, cardiovascular disease, osteoporosis, and chronic degenerative diseases [1–3]. Furthermore, legumes have a low glycemic index, ranging from 10 to 40. In general, a serving of legumes (~ 100 g fresh weight) provides 115 cal, 20 g of carbohydrates, 7–9 g of fiber, 8 g of protein, and 1 g of fat [4].

Legumes, and in particular soy, constitute an important part of the diet for the majority of Asian population, and many studies have investigated their potential health promoting effects. Soybeans and soy-based food products contain uniquely high isoflavone content ~ 1–3 mg isoflavones/g protein, and one serving of traditional soy foods provides ~ 25–40 mg isoflavones [5] as compared to other commonly consumed plant foods. In fact, USDA Database on the isoflavone content of the selected foods [6] has reported very high total isoflavone content from soybean and soy-based products as compared to other vegetables and foods. Of the 114 commonly consumed vegetables of Europe analyzed, the foods derived from soy contained isoflavone concentration (500–1400 mg daidzein and genistein/kg) at least two orders of magnitude higher than the next richest isoflavone food (raw mung beansprouts; 6 mg/kg) and several orders of magnitude higher concentration than the non-leguminous sources [7]. Consumption of an isoflavone rich soy diet has been linked to improved health outcomes in a number of studies [5, 8–10]. These health benefits may arise in part due to the presence of various isoflavonoid components such as daidzein, genistein, and glycitein which were characteristic of soy possessing numerous biological functions [5]. These isoflavonoids are found in conjugated form with either glucose or 6″-*O*-malonyl- or 6″-*O*-acetylglucose in plants [11]. Daidzein is further metabolized by intestinal gut bacteria to equol, *O*-desmethylangolensin (*O*-DMA), dihydrodaidzein, and *cis*-4-OH-equol, while genistein is further metabolized to dihydrogenistein and 6’-OH-*O*-DMA [12, 13]. The beneficial effects of soy seem to be related to the combination of these compounds and not any one in particular. For example, daidzein and genistein were reported to have a synergistic effect on inhibiting cell proliferation and inducing apoptosis of prostate cancer cells [14]. In addition, these compounds and equol were also proposed to be antiestrogenic, antioxidative, and anticarcinogenic and may protect against chronic diseases such as hormone-dependent cancer, cardiovascular diseases, and osteoporosis [15–19]. However, it is worth noting that a few studies considering estrogenic effects of dietary soy phytoestrogens have demonstrated that the isoflavones promote growth of ER-positive breast cancer cells under both in vitro as well as in vivo conditions [20–22]. With respect to dietary pulses, a sub-group of legumes, dietary intake has been associated with reduced chronic disease risks for cardiovascular disease and cancer [23], as well as with improvements in intermediate cardiovascular disease risk factors, such as blood pressure, dyslipidemia, glycemic control, and weight management [23–28]. Pulses also provide a valuable means of lowering the glycaemic-index (GI) of the diet [28].

Due to the health benefits of legumes, there is a growing interest in assessing their dietary intake across different populations. In order to achieve this to a high standard, it is pertinent that we have more accurate and reliable assessment tools to monitor their intake. The classical approaches of data collection tools such as food frequency questionnaires (FFQ), food dairies, and 24-h dietary recalls are associated with a number of errors [29–33]. Hence, there is a growing need for more objective measures of intake, and biomarkers have emerged as having great potential in this field. Such biomarkers should be able to reflect the differences in dietary intakes across a number of population types [34].

The objective of this paper was to perform a systematic review of the literature and summarize the information from observational and human intervention studies on the biomarkers of legumes intake and also evaluate the validity, reproducibility, and sensitivity of the proposed markers that could potentially be useful indicators of legume consumption.

## Search methodology

**Fig. 1:**
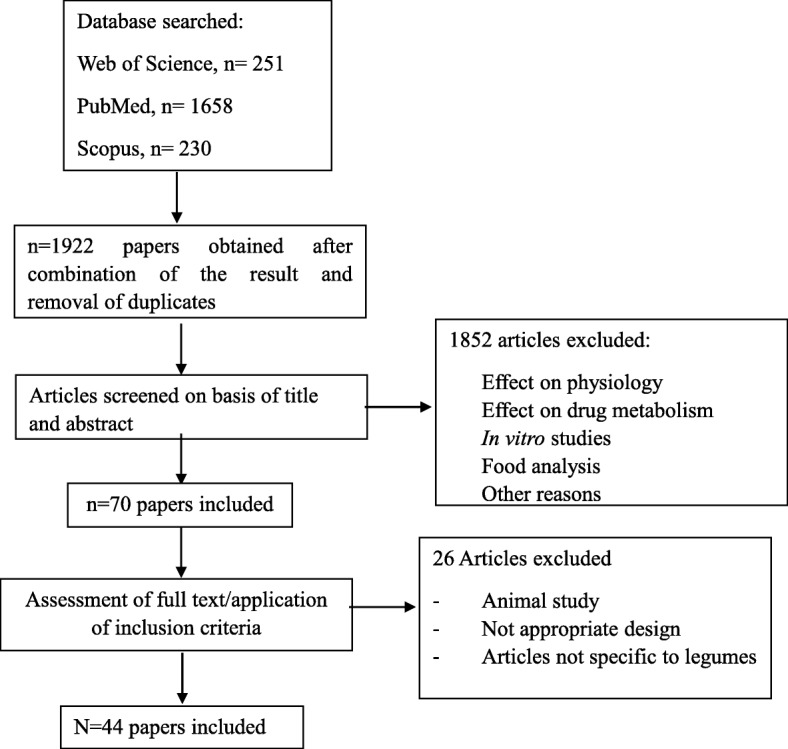
Flow diagram of the study selection

The reviewing process made use of elements of Preferred Reporting Items for Systematic Reviews and Meta-analyses (PRISMA) statement [35], which were relevant for a search for literature on biomarkers. The search methodology was also followed in accordance with the guidelines for biomarkers of food intake reviews (BFIRev) [36]. The search process included results until 16 February 2018. In brief, original research papers and reviews were searched in three databases (PubMed, Scopus, and ISI Web of Knowledge) using combinations of the grouped search terms (legume OR bean OR pea) AND (biomarker* OR marker* OR metabolite* OR biokinetics OR biotransformation) AND (trial OR experiment OR study OR intervention) AND (human* OR men OR women OR patient* OR volunteer*) AND (urine OR plasma OR serum OR blood OR excretion) AND (intake OR meal OR diet OR ingestion OR consumption OR eating OR drink*). The research was limited to papers in English language, while no restriction was applied for the publication dates. The research papers identifying or using potential biomarkers of intake for the foods were selected by one or more skilled researcher from the list of retrieved references taking into consideration inclusion criteria with literature focused on intervention studies in which participants consume known amount of specific foods and biological samples collected and also population studies. The exclusion criteria include literature reported with reference to effect of physiology, drug metabolism, in vitro studies, food analysis studies, animal studies, inappropriate study designs, and non-specific articles to legumes food group [36] as outlined in Fig. 1.

Considering the list of discriminating metabolites obtained from the primary search, a secondary search was performed to identify other foods containing the same biomarkers or precursors and to determine the apparent specificity of the compound of interest. In this second step, PubMed, Scopus, and Web of Science were used as search platforms and the compounds checked for their specificity were genistein, daidzein, dihydrogenistein, dihydrodaidzein, enterodiol, enterolactone, matairesinol, *O*-DMA, glycitein, kaempferol, dimethylamine, glutamine, 3-methylhistidine, trigonelline, pipecolic acid, indolepropionate, *S*-methylcysteine, and *N*-acetyl-ornithine and their synonyms (Additional file 1: Table S1). For each of these potential biomarkers identified, an additional search was conducted using the following search criteria (“the name and synonyms of the compound” OR “the name and synonyms of any parent compound”) AND (biomarker* OR marker* OR metabolite* OR biokinetics OR biotransformation) AND (trial OR experiment OR study OR intervention) AND (human* OR men OR women OR patient* OR volunteer*) AND (urine OR plasma OR serum OR blood OR excretion) AND (intake OR meal OR diet OR ingestion OR consumption OR eating OR drink*).

The validity of candidate biomarkers obtained from the above search was further assessed through a consensus-based procedure which evaluated a set of the most important criteria for systematic evaluation of biomarkers of food intake. The scoring scheme presented in this review aimed to address criteria which include plausibility, dose-response, time-response, robustness, reliability, stability, analytical performance, and inter-laboratory reproducibility (Table 2). A detailed explanation of critical assessment of the criteria chosen for biomarker validation was previously presented [37].

## Results and discussion

The literature search performed identified a total of 2139 articles from the three databases, and a flow diagram of the study selection is represented in Fig. 1. A total of 1922 articles were obtained after removal of duplicates using Endnote X7.4. Of these, a total of 70 articles were selected after screening on the basis of title and abstract. Exclusion criteria for the remaining 1852 articles included the following: effect on physiology, effect on drug metabolism, in vitro studies, food analysis, and other articles related to antioxidant markers, disease/health markers, oxidative stress markers, articles not relevant to intake biomarkers, and animal studies. Full texts of the 70 papers were downloaded and assessed further for exclusion/inclusion criteria. Exclusion criteria at this stage included animal studies, inappropriate study design and articles not specific to legume intake. In total, 44 articles were retained and used for the development of the tables. Table 1 provides a summary of the selected studies, including the candidate biomarkers for legumes/beans/peas intake identified through this search process.

**Table 1:** List of reported putative legume biomarkers of intake

Dietary factor	Subject	Study design	Number of subjects	Analytical method	Sample type	Discriminating metabolites/candidate biomarkers	Reference
Soy beans/tofu	Human (M/F)	Cross-sectional study	98	GC-MS	Urine (24 h)	Genistein Daidzein *O*-DMA	[41]
Soy-based foods	Human (F)	Cross-sectional study	102	HPLC	Urine (24 h)	Genistein Daidzein *O*-DMA Glycitein	[44]
Soy-based foods	Human (F)	Cross-sectional study	60	HPLC	Urine	Genistein Daidzein *O*-DMA Glycitein	[39]
Soy-based foods	Human (M/F)	Cross-sectional study	147	HPLC	Urine (spot)	Genistein Daidzein *O*-DMA Glycitein Equol	[38]
Soy protein	Human (M/F)	Cross-sectional study	100	HPLC-MS	Urine (overnight)	Genistein Daidzein Equol	[40]
Soy-based foods	Human (F)	Cross sectional study	27/451	GC-MS	Urine (24 h, overnight)	Genistein Daidzein	[43]
Soy milk	Human (F)	Observational study	159	MS	Urine (24 h)	Equol	[87]
Soy-based foods	Human (F)	Cross section	363	GC-MS	Urine (2 Overnight, 48 h apart)	Genistein Daidzein *O*-DMA Equol	[42]
Soy-based foods	Human (M/F)	Cross-sectional study	77	LC-MS	Plasma (fasting)	Genistein Daidzein	[46]
Soy-based foods	Human (F)	Cross-sectional study	80	Flouroimmuno assay	Plasma	Genistein Daidzein	[45]
Soy-based foods	Human (F)	Cross-sectional study	1823	LC-coularray, LC-MS	Serum (non-fasting)	Genistein Daidzein	[49]
Soy-based foods and supplements	Human (F)	Cross-sectional study	96	LC-MS	Plasma (fasting)	Genistein Daidzein	[47]
Soy-based foods	Human (F)	Case Control Study	97 (Cases) 97 (Control)	Isotope Dilution Electrospray Tandem Mass Spectrometry	Plasma (randomly timed)	Genistein Daidzein Equol Dihydrogenistein Dihydrodaidzein	[48]
Soy bean products	Human (M)	Intervention study	17	GC-MS	Urine (24 h)	Genistein Daidzein *O*-DMA Equol Enterodiol Enterolactone Matairesinol	[56]
Soy-based foods	Human (F)	Intervention study	18	HPLC-MS	Urine	Genistein Daidzein *O*-DMA Glycitein Equol	[90]
Soymilk powder	Human (F)	Acute study	12	HPLC	Urine (0–24 h after intake)	Genistein Daidzein	[19]
Soy bean powder (Kinako)	Human (M)	Acute study	7	GC-MS	Urine (0–24 h)	Genistein Daidzein *O*-DMA Equol	[89]
Soy nuts	Human (F)	Acute study	10	GC-MS	Urine (0–12 h for 5 days)	Genistein Daidzein Equol	[68]
Soymilk-based beverages	Human (M/F)	Acute study	12	LC-MS	Urine (0–48 h)	Genistein Daidzein Glycitein	[59]
Soy isoflavones	Human (F)	Intervention study (30 days) Intervention study (60 days)	27 12	ELISA	Urine (24 h)	Genistein Daidzein Equol	[71]
Soy capsules (Phytosoya)	Human (M)	Acute study	12	ELISA	Urine (0-24 h)	Genistein Daidzein Equol	[78]
Soy-based foods	Human (M/F)	Intervention study	20	GC-MS	Urine (24 h)	Genistein Daidzein *O*-DMA Equol Enterodiol Enterolactone	[52]
Soy-based foods	Human (F)	Intervention study	43	LC-MS	Urine (overnight)	Genistein Daidzein Equol	[81]
Soy-based foods	Human (F)	Intervention study	350	LC-MS	Urine (overnight, spot)	Genistein Daidzein *O*-DMA Equol Glycitein Dihydrogenistein Dihydrodaidzein	[58]
Soy flour	Human (M/F)	Acute study	12	GC-MS	Urine (0–24 h on 0 days, 3 days, 4 days, 5 days)	Genistein Daidzein *O*-DMA Equol Glycitein Dihydrodaidzein Enterolactone	[50]
Soy protein beverage	Human (M/F)	Acute study	60	GC-MS	Urine (0–24 h on 4 days)	Equol Daidzen Genistein *O*-DMA	[80]
Soy milk, Miso soup	Human (F)	Intervention study	21	LC-MS	Urine (overnight, spot) for 6 days	Daidzein Genistein Equol	[61]
Soy milk	Human (F)	Acute study	6	GC-FID	Urine (1–4 days, 16–18 days, and 30–32 days)	Daidzein Genistein Equol	[79]
Soy-based foods	Human (F)	Intervention study	350	LC-MS	Urine (overnight, spot)	Equol Daidzein	[84]
Soy-based diet	Human (F)	Intervention study	256	HPLC; LC-MS/MS	Urine (overnight)	Total isoflavone excretion	[73]
Commercial soy preparation	Human (M)	Intervention study	7	GC-MS	Urine (0–24 h)	Daidzein Daidzein glucoside Dihydrodaidzein *O*-DMA Equol	[63]
Soy flour-based meal	Human (M)	Acute study	6	HPLC	Plasma (0–35 h after intake)	Genistein Daidzein	[67]
Soy extract capsule/Soy beverage	Human (F)	Acute study	12	HPLC	Plasma (0–32 h after intake)	Genistein Daidzein	[69]
Soymilk powder	Human (F)	Acute study	12	HPLC	Plasma (0–24 h after intake)	Genistein Daidzein	[19]
Soy protein powder	Human (M)	Intervention study	20	GC-MS	Plasma	Genistein Daidzein	[54]
Soy bean powder (Kinako)	Human (M)	Acute study	7	GC-MS	Plasma (0–72 h after intake)	Genistein Daidzein *O*-DMA Equol	[89]
Soy nuts	Human (F)	Acute study	10	HPLC-MS GC-MS	Serum (0–48 h)	Genistein Daidzein Equol	[68]
Soymilk-based beverages	Human (M/F)	Acute study	12	LC-MS	Serum (0–24 h)	Genistein Daidzein Glycitein	[59]
Soy isoflavones	Human (F)	Intervention study Intervention study	27 12	ELISA	Plasma (fasting)	Genistein Daidzein Equol	[71]
Soy capsules (Phytosoya)	Human (M)	Acute study	12	ELISA	Plasma (0–48 h)	Genistein Daidzein Equol	[78]
Soy-based foods and supplements	Human (F)	Cross-sectional study	96	LC-MS	Plasma (fasting)	Genistein Daidzein	[47]
Soy bean extracts	Human (M/F) Human (M)	Acute study Intervention study	16 8	HPLC HPLC	Plasma (0-24 h after intake) Plasma (non-fasting)	Genistein Daidzein	[62]
Soy-based foods	Human (F)	Intervention study	350	LC-MS	Plasma (baseline, every 6 months for 2.5 years)	Genistein Daidzein *O*-DMA Equol Glycitein Dihydrogenistein Dihydrodaidzein	[58]
Soy-based foods	Human (F)	Intervention study	350	LC-MS	Plasma (baseline, every 6 months for 2.5 years)	Equol Daidzein	[84]
Commercial Soy isoflavone supplements	Human (F)	Acute study	19	GC-MS	Plasma (0–48 h)	Genistein Daidzein Genistin Daidzin	[64]
Commercial soy preparation	Human (M)	Intervention study	7	GC-MS	Plasma (0–48 h)	Daidzein Daidzein glucoside Dihydrodaidzein *O*-DMA Equol	[63]
Soy bean powder (Kinako)	Human (M)	Acute study	7	GC-MS	Feces (24-72 h)	Genistein Daidzein *O*-DMA Equol	[89]
Dry beans	Human (M/F)	Observational study	106	LC-MS; GC-MS	Serum (fasting)	Pipecolic acid *S*-Methyl cysteine *N*-Acetylornithine Trigonelline Indole propionate	[94]
Dry beans	Human (M) Mouse (M)	Intervention study Intervention study	46 12	LC-MS; GC-MS LC-MS; GC-MS	Serum (fasting) Serum	Pipecolic acid *S*-Methyl cysteine *N*-Acetylornithine Trigonelline Indole propionate Pipecolic acid *S*-Methyl cysteine *N*-Acetylornithine	[94]
Beans	Human (M/F)	Acute study	7	HPLC	Urine (0–24 h after intake)	Kaempferol	[95]
Green peas	Human (M/F)	Intervention study	9	NMR	Urine (0–3 days)	Trigonelline	[98]
Pulses	Human (M/F)	Observational study	50	NMR	Spot urine	Dimethylamine Glutamine 3-Methylhistidine	[97]

### Cross-sectional studies reporting on isoflavones and their metabolites as markers of soy intake

Examination of cross-sectional studies revealed that a number of studies investigated the isoflavones and their metabolites such as genistein, daidzein, glycitein, and *O*-DMA in biological samples such as urine and blood (serum and plasma) following the consumption of soy or soy-based foods (Table 1).

A study conducted in 147 Singaporean Chinese with spot urine samples demonstrated a statistically significant, dose-dependent association between frequency of overall soy intake and levels of urinary daidzein (*p* = 0.03) and sum of urinary daidzein, genistein, and glycitein (*P* = 0.04) [38]. A dose-response relationship was also observed between dietary soy consumption and urinary excretion rates of daidzein, genistein, and glycitein as well as with total isoflavones (*p* ≤ 0.05) in Chinese women (*n* = 60) [39]. There were also positive correlations established between urinary isoflavones excretion and the amount of soy food, soy protein, and soy isoflavones intake (*r* = 0.50, *p* < 0.001, *r* = 0.53, *p* < 0.001 and *r* = 0.54, *p* < 0.001, respectively). Similarly, another study examining a Western population (*n* = 100) showed significant correlations between soy protein intake from 24-h recalls with daidzein (*r* = 0.72, (CI) 0.43, 0.96), genistein (*r* = 0.67, (CI) 0.43, 0.91), and total isoflavones (*r* = 0.72, (CI) 0.47, 0.98) [40]. Additionally, between FFQs and urinary excretion, the correlations were (*r* = 0.50, (CI) 0.32, 0.65), (*r* = 0.48 (CI) 0.29, 0.61) and (*r* = 0.50 (CI): 0.32, 0.64) for daidzein, genistein, and total isoflavones, respectively [40]. Significant correlations were reported between intake of soy foods through FFQ and 5-day diet records, with urinary genistein (*r* = 0.40, *p* = 0.0001), *O*-DMA (*r* = 0.37, *p* = 0.0002), daidzein (*r* = 0.34, *p* = 0.0007), and the sum of isoflavones (*r* = 0.39, *p* = 0.0001) in US men and women (*n* = 98) [41]. In another US population study, positive correlations were demonstrated between self-reported soy intake and excretion of urinary isoflavones (*r* = 0.52, *p* < 0.001 for dietary recall and *r* = 0.29, *p* < 0.01 for FFQ) [42]. In a US study (*n* = 451 women) [43], 24-h urine sample measures were shown to be strongly correlated with overnight urine excretion for daidzein (*r* = 0.84) and genistein (*r* = 0.93). The 24-h urine sample measures were also correlated with soy food questionnaire (SFQ) estimates of daidzein (*r* = 0.48) and genistein (*r* = 0.54) intake [43]. A significant correlation between isoflavones measured in an overnight urine and soy protein intake estimated by self-reported intake (dietary questionnaire) was also established in multiethnic population (*n* = 102), both in the previous 24 h (*r* = 0.61, *p* < 0.0001) and in the past year (*r* = 0.32, *p* < 0.0012) [44]. Overall, significant correlations between soy intake and urinary isoflavones excretion either in spot, overnight, or 24-h urine samples were demonstrated suggesting that these compounds have the potential to serve as dietary biomarkers.

While the above studies have focused on isoflavone levels in urine, there was also evidence to support relationships in plasma and serum. A study of four groups of 20 premenopausal British women (*n* = 80) demonstrated significant correlations between dietary total soy intake estimated by FFQ and food diaries with plasma daidzein (*r* = 0.74–0.78, *p* < 0.001) and genistein (*r* = 0.73–0.78, *p* < 0.001) [45]. Similarly, significant correlations were reported between genistein and daidzein intakes as determined by soy FFQ with plasma concentrations (*r* = 0.53 and 0.45) respectively in a Western population group (*n* = 77) [46]. Furthermore, similar results were found in US postmenopausal women (*n* = 96) with correlations varying from 0.35 to 0.43 depending on the dietary intake instrument [47]. A statistically significant (*p* = 0.002) threefold difference in mean plasma levels of total isoflavones was observed between women with high and low soy isoflavone intake levels as determined from FFQ [48]. For the correlation between serum isoflavones levels and soy intake, a significant linear trend (*p* < 0.01) was observed in serum isoflavones (daidzein and genistein) concentrations across increasing categories of soy food consumption estimated by FFQ in Asian women (*n* = 1823) [49].

In summary, the cross-sectional studies demonstrated that there were positive correlations between soy intake with urinary, plasma, and serum isoflavones levels, mainly daidzein and genistein, in different population groups.

### Acute and intervention studies reporting relationships between soy intake and isoflavones and their metabolites

#### Soy isoflavones

The literature search identified a number of intervention studies which focused on soy-based diets and isoflavonoid excretion in different population groups (Table 1). Various acute studies have reported increased isoflavonoid concentrations in blood and urine following consumption of soy-based foods (Table 1). To understand the metabolic fate of dietary isoflavones in humans, a study examined 24-h urines from 12 healthy Caucasian male and female participants following 3 days of soy challenge: the urinary isoflavone levels (genistein, daidzein, glycitein) peaked more than 3.8-fold and returned to basal levels by day 4, while the major urinary metabolites (*O*-DMA, equol, 6-hydroxy-*O*-DMA, dihydrodaidzein) demonstrated a more significant increase of over 5- to 40-fold and progressively fell over days 4 and 5 [50]. This marked variation among the major urinary isoflavonoid metabolites may reflect variability in an individual’s ability to ferment isoflavones and the fat content of the diet [51]. A randomized controlled crossover feeding study demonstrated that the urinary excretion of total isoflavones significantly increased with soy diet (normal basal diet plus 100 g tofu and 45 g of soy protein isolate served per day) consumption (26.01 ± 2.30 μmol/day) as compared to the vegetable free (0.75 μmol/day), carotenoid (0.51 μmol/day), and cruciferous vegetable diet (1.03 μmol/day) [52]. Overall, the results from this study provide information on the utility of urinary isoflavones as biomarkers of soy intake.

In another study, the urinary recovery of daidzein levels were significantly higher than genistein (*p* < 0.001), while the plasma concentrations of both isoflavones did not differ significantly (*p* > 0.1) after single doses of 0.7, 1.3, and 2.0 mg isoflavones/kg body weight in soybean milk [19]. Subsequently, a randomized, double-blind, crossover study involving four 9-day soy protein beverage supplementation periods established a positive dose-response between urinary isoflavones excretion and soy intake (*p* = 0.0001) with no significant difference between equol excretors and non-excretors [53].

A study examining the repeated intake of consuming soy protein powder of about 60 g/day in a controlled intervention trial for over a period of 28 days demonstrated that the plasma isoflavone levels markedly increased on day 28 compared to day 0 with no change in the control group on a casein supplement diet [54]. This result was in agreement with other related studies, which demonstrated an increase in isoflavonoid excretion following a soy challenge [19, 50, 55, 56].

Glycitein is a soy isoflavonoid which constitutes 5 to 10% of the total isoflavones in the soy beans [57]. A few cross-sectional studies have reported urinary excretion of glycitein [38, 39, 44], but the excretion levels are low compared to genistein and daidzein [58]. Maximum serum concentration for glycitein attained after ingestion of aglycone and glucosidic forms of soy beverage did not differ significantly (0.07–0.09 μmol/L), and maximum urinary excretion was reported to be ~ 3 μmol [59]. Similarly, maximum glycitein plasma concentration of ~ 200 ng/ml and maximum glycitein urinary concentration of 11,000 ng/ml were reported in a bioavailability study [60]. A large soy intervention trial demonstrated a three- to fourfold increase in glycitein levels in plasma, overnight urine, and spot urine was observed in the soy group compared to the placebo. However, the magnitude of increase was smaller compared to major soy isoflavones genistein and daidzein [58].

Several studies have also reported differences in isoflavone excretion with respect to the type of soy foods with most interest in the difference between fermented and non-fermented sources. A study comparing the effects of fermented and non-fermented soy product consumption demonstrated that the urinary isoflavone recovery of genistein and daidzein was higher (*p* < 0.002) when the subjects consumed tempeh (fermented) compared to the soybean pieces diet [56]. This suggests that the fermented products, due to the hydrolysis of isoflavone glucosides to their corresponding aglycones could have increased availability of the isoflavones. In contrast, urinary isoflavonoid excretion showed no significant difference upon consumption of soymilk (non-fermented) compared to miso soup (fermented) (*p* = 0.87) [61].

Similarly, a study comparing the effects of fermented and non-fermented soy product consumption demonstrated that the plasma concentrations of genistein and daidzein were more than twice and five times higher (*p* < 0.05) when subjects consumed fermented soybean extract compared to non-fermented soybean extract [62]. Similar results were reported for total isoflavones with higher serum isoflavone concentrations (~ 2 μmol/L) attained with fermented soymilk ingestion compared to non-fermented soymilk (~ 0.94 μmol/L) [59]. In contrast, reports following ingestion of commercial soy supplements have reported that plasma isoflavone concentrations were higher following ingestion of glucosidic forms compared to aglycone forms [63, 64]. However, it should also be noted that hydrolysis of isoflavone glycosides to their corresponding aglycones did not seem to alter plasma concentrations in some studies [65, 66].

Overall, the urinary excretion levels and plasma concentrations of soy isoflavones were reported to have variable responses to the consumption of isoflavone aglycone- and isoflavone glucoside-rich foods.

#### Pharmacokinetics of isoflavones

A number of studies investigated the pharmacokinetic behavior of isoflavones following soy intake. In all studies, the shapes of the plasma appearance and disappearance curves with respect to time exhibited biphasic pattern as a result of enterohepatic circulation of the compounds. Peak plasma concentration of isoflavones following consumption of a soy-based meal was reported for genistein at 8.42 ± 0.69 h (*t*_1/2_ = 5.7 ± 1.3 h) and daidzein at 7.42 ± 0.74 h (*t*_1/2_ = 4.7 ± 1.1 h) [67]. Similarly, genistein was reported as having a longer half-life (8.36 h) compared to daidzein (5.79 h) following consumption of 60 g of kinako (baked soybean powder) [59]. Peak serum concentrations of daidzein and genistein on average were attained at 6.9 ± 0.7 h and 6.5 ± 1.0 h, respectively, and their corresponding elimination half-lives were reported as 8 and 10.1 h, respectively, following consumption of 10, 20, or 40 g of soy nuts [68]. A curvilinear relationship was established between bioavailability for daidzein and genistein and the increased amount of soy nuts intake. This suggests a decrease in serum concentrations measured at increasing dosage levels. The same study revealed that most of the excreted urinary isoflavones were eliminated within the first 2 days following consumption of soy nuts at different doses. However, the urinary daidzein excretion decreased from approximately 63 to 44%, while the urinary genistein excretion decreased from 25 to 15% upon increased amount of soy nuts intake from 10 to 40 g. This non-linear pharmacokinetic behavior over a dose range reveals that optimum steady state isoflavone concentrations can be achieved by multiple intakes of soy foods at regular intervals of time than a single high dosage soy product [68]. Similarly, a randomized two-phase crossover study reported peak plasma concentrations of daidzein and genistein on average attained at 6.08 h and 6.37 h, respectively, and their corresponding half-lives were 7.17 h and 7.7 h, respectively [69]. Typically in all of the studies, urinary recovery of genistein and daidzein is complete within 24–36 h [70]. According to the evidence obtained from the literature, both urine and plasma can be considered suitable biofluids to measure soy intake.

#### Chronic ingestion of soy isoflavones

A study in post-menopausal women who chronically ingested the commercial soy-based preparation Prevastein (46.19 g of total isoflavones expressed in the aglycone form per 100 g of preparation) for 30 and 60 days demonstrated that the urinary and plasma concentrations of genistein, daidzein, and equol remained constant from day 15 until the end of experimental period [71]. The data suggest that chronic ingestion could lead to a saturation point and optimum steady state biofluid concentrations can be achieved consistently with adequate intake of soy foods or supplements. However, from a biomarker view point, this may indicate a limitation for estimation of high intakes [68, 71]. An intervention study in young girls who consumed either one daily serving of soymilk (8.5 oz) or soy nuts (1 oz) for 8-week period demonstrated that urinary excretion of soy isoflavones increased by almost sixfold from baseline (23.3 to 142 nmol/mg creatinine). This finding was also reported to be consistent with the 3-day food record which showed a significant increase in isoflavone intake (5.4 to 32.6 mg/day) during the intervention period [72].

While there are many studies focused on concentration levels of isoflavones present in the biofluids following consumption of a range of soy foods, these studies have limited number of subjects and some of the studies lack repeated collection of biofluids. A large randomized, double-blind soy intervention trial with 350 postmenopausal women for 3 years established high correlations between isoflavone measurements of overnight urine, spot urine, and plasma with Pearson correlations ranging between 0.60 and 0.94 [58]. All three matrices showed significantly high isoflavone quantitative differences of up to 3–19-fold between placebo and soy group and also highly significant correlations between mean isoflavone values and soy doses, but not in the placebo group. In another two randomized soy trials conducted among 256 premenopausal women consuming high (~ 50 mg isoflavones/day) and low (~ 10 mg isoflavones/day) soy diets, urinary isoflavonoid excretion significantly correlated to dietary isoflavone intake (*r* = 0.51, AUC = 0.85; *p* < 0.0001) [73]. Overall, these studies provide further support for the use of isoflavones as biomarkers of dietary soy intake.

Soybeans are consumed mainly as processed soy products such as tofu, milk, nuts, and protein isolate powder. The influence of soy food matrix and the effect of industrial processing has resulted in varied isoflavone contents in soy-based products [74, 75]. The varied degree of processing conditions has also influenced the metabolism, pharmacokinetics, and bioavailability of soy isoflavones [55, 64, 76, 77]. However, our review concentrated on covering information on potential biomarkers obtained from soy and soy-based food products rather than trying to understand the influence of abovementioned conditions on the bioavailability of biomarkers. Furthermore, it should be noted that the biomarkers cannot distinguish between food and supplement sources.

#### Soy isoflavone metabolites

A number of soy isoflavone metabolites are found in the circulation. The following section will highlight the key isoflavone metabolites found in the literature review.

Equol is a major isoflavonoid estrogen metabolite produced from daidzein by gut microbiota and is produced by ~ 30–40% of individuals after a soy challenge (named “equol producers”) [70, 78]. A chronic soy exposure study demonstrated that the urinary recovery of equol increased by 3–100-fold (*p* < 0.05) over 4 weeks of daily soy ingestion [79]. Similarly, a study examining the prevalence of equol excretion in both male and female individuals revealed that 35% of the participants among the 60 were found to excrete equol following soy protein beverage consumption after 3 days [80]. However, the common isoflavones excreted after ingestion of soy-based foods such as daidzein, genistein, and *O*-DMA was similar between equol excretors and non-excretors in both men and women [80]. A similar study demonstrated an increase in equol production in older women, while the total excretion of isoflavones remained the same after a standardized dose of soy milk among three generations of American-Japanese women [81]. The differences in excretion could in part be attributed to differential gut microbiota composition with age and differential habitual dietary compositions [51, 82, 83]. Equol production was studied over a period of 3 years, and results indicated a high intraindividual variability [84]. However, in other studies, equol production was reported to be relatively stable over time [85, 86]. Such differences in equol production could be due to dietary factors such as minor differences in intake of micronutrients [87], but further research needs to be done to consider other factors responsible for variation in equol production.

*O*-DMA is an isoflavonoid estrogen metabolite formed when daidzein is metabolized to dihydrodaidzein by intestinal bacteria in the large intestine and further undergoes ring cleavage [88]. Urinary excretion of *O*-DMA was reported to be generally higher when subjects consumed soy-based foods [50, 52, 56, 53, 89]. A randomized crossover study also demonstrated that *O*-DMA appears in plasma after ~ 6–8 h post-consumption of daidzein rich soy isoflavone preparation and also observed almost a twofold increase after ingestion of pure daidzein glucoside compared to the aglycone form. Urinary excretion levels of *O*-DMA were also two times higher following ingestion of the glucoside form compared to aglycone form [63]. In a large soy intervention trial, ~ 10-fold increase in *O*-DMA levels in plasma, overnight urine, and spot urine was observed in the soy group fed with soy beverage powder and soy bars compared to the placebo fed with protein isolates and bars with no isoflavone content [58]. A lower urinary isoflavone excretion value for *O*-DMA was reported in the equol excretors probably due to conversion of daidzein to equol [90].

Additionally, some studies have reported the urinary dihydrogenistein and dihydrodaidzein (intermediate products of soy isoflavone metabolism) levels post-consumption of soy-based foods [50, 91, 92]. Consumption of soy compared to placebo resulted in ~ 4–7 fold higher dihydrogenistein and dihydrodaidzein [58]. While these soy isoflavone metabolites reveal interesting metabolic information, it remains to be determined if they are useful as biomarkers of soy intake.

#### Lignan phytoestrogens

A few studies have reported either low or no association of lignans in biofluids to consumption of soy or soy-based foods. The urinary excretion of lignans enterodiol and enterolactone was reported to be low after consumption of soy rich diet [93] and fermented and unfermented soy products [56]. The enterolactone levels were found to remain unaffected following a soy challenge [50]. Furthermore, no differences in excretion levels of lignans were found following soy-based diet and basal diet consumption [52]. Subsequently, the urinary excretion levels of lignans are high following consumption of other sources of lignans such as cruciferous vegetable diets (3.86 ± 0.21 μmol/day) as compared to soy diet (0.84 ± 0.21 μmol/day) [52], and hence, lignans cannot be considered as markers of soy consumption.

### Studies relating pulses intake to metabolites in biofluids

While most of the studies retrieved are focused on soybeans, there are a few studies which proposed metabolites related to pulses intake. A randomized controlled crossover human feeding study involving 46 middle-aged men following consumption of a high dry bean-enriched diet (250 g/day) for 4 weeks led to elevated serum levels of pipecolic acid, *S*-methyl cysteine, *N*-acetylornithine, trigonelline, and indole propionate [94]. Based on a further study in which participants self-reported their dry bean intake, only pipecolic acid and *S*-methyl cysteine reflected dry bean consumption. Therefore, these two metabolites were proposed as useful markers of dry bean consumption [94]. In a different study, maximum peak urinary excretion of kaempferol was observed after 2–8 h following consumption of cooked beans (*Phaseolus vulgaris* L.) [95]. The average excretion was 6.1% and 5.4% of kaempferol dose for males and females respectively. However, although the excretion profiles were similar between subjects, a 6.72-fold inter-individual variation in excretion concentrations was reported, which was ascertained to variations in intestinal physiology [95]. A study examining urinary exposure markers of a wide range of individual foods and food groups revealed that the most probable food exposure marker for green beans was an unsaturated aliphatic hydroxyl-dicarboxylic acid [96]. An observational study aimed to characterize the urinary metabolomic fingerprinting revealed glutamine, dimethylamine, and 3-methylhistidine as candidate biomarkers of pulse consumption [97]. A recent study identified trigonelline as urinary biomarker of pea intake although reported to be non-specific marker of pea consumption [98]. Notwithstanding the substantial research performed on soy isoflavones, further research still needs to be performed in order to identify potential biomarkers of pulses and pulse-based foods in general.

### Overall usefulness of the biomarkers

The assessment of data presented in Table 2 and the secondary search performed revealed that the compounds genistein and daidzein are present in very high concentrations in soybeans and in moderate concentrations in legume-based vegetables such as beansprouts, chickpeas, lentils, fava bean, and roots of kudzu wine [7, 99]. They are also present in lower concentrations in other fruits and vegetables such as potato, tomato, cabbage, turnip, pumpkin, and asparagus [7]. Although there are some reports of excretion of daidzein and genistein and their precursors following consumption of red clover [100], the higher concentrations following soy consumption make them highly specific markers of soy consumption as evidenced in the published studies. Moreover, genistein and daidzein were observed as having a dose-dependent relationship with soy intake in various observational studies [38, 39, 44, 46, 47]. Dose-response effects after a single bolus ingestion of three different doses of soy isoflavones were also reported [68]. The time-response relationship explaining the elimination half-life of genistein and daidzein [64, 89] as well as kinetics of repeated intake [71] were also highlighted in this review. Both genistein and daidzein are also proven to be stable in urine and plasma at − 20 °C for almost 3 months [101] and various quantification methods using LCMS and GCMS platforms for genistein and daidzein have been developed. The recovery %, limit of detection, and sensitivity and specificity of genistein and daidzein were reported in different analytical methods [102]. A multi-laboratory validation study across seven different laboratories proposed to determine and quantify the isoflavone content in three soybean varieties showed a satisfactory interlaboratory precision [103]. However, there are no reports of individual isoflavones reported for interlaboratory reproducibility. From a robustness point of view, while the cross-sectional studies have demonstrated significant associations with soy intake, a number of potential confounding factors such as gut microbial populations, intestinal transit time, and gender were identified. Furthermore, data also exists which demonstrates that plasma isoflavone concentrations were positively associated with age, fiber consumption, servings of fruits and vegetables, and dietary supplements [98]. Consequently, additional research efforts are needed to establish more clearly the relationship between the biomarkers and habitual diet in larger population-based studies and after intake of complex meals in intervention studies with more number of subjects.

**Table 2:** Possible scoring scheme for legume intake biomarker validity

Food item	Metabolites	Biofluid locations	Questions							
			1	2	3	4	5	6	7	8
Soy	Genistein	Urine	Y	Y	Y	N	N	Y	Y	U
		Plasma/serum	Y	Y	Y	N	N	Y	Y	U
	Dihydrogenistein	Urine	N	U	U	N	N	U	Y	U
		Plasma/serum	N	U	U	N	N	U	Y	U
	Dihydrodaidzein	Urine	N	U	U	N	N	U	Y	U
		Plasma/serum	N	U	Y	N	N	U	Y	U
	Enterodiol	Urine	N	U	U	N	N	U	Y	U
		Plasma/serum	N	U	U	N	N	U	Y	U
	Enterolactone	Urine	N	U	U	N	N	U	Y	U
		Plasma/serum	N	U	U	N	N	U	Y	U
	Matairesinol	Urine	N	U	U	N	N	U	Y	U
		Plasma/serum	N	U	U	N	N	U	Y	U
	Daidzein	Urine	Y	Y	Y	N	N	Y	Y	U
		Plasma/serum	Y	Y	Y	N	N	Y	Y	U
	Equol	Urine	N	U	Y	N	N	U	Y	U
		Plasma/serum	N	U	Y	N	N	U	Y	U
	O-desmethylangolensin	Urine	N	U	U	N	N	U	Y	U
		Plasma/serum	N	U	Y	N	N	U	Y	U
	Glycitein	Urine	Y	U	Y	N	N	U	Y	U
		Plasma/serum	Y	U	Y	N	N	U	Y	U
Pulses	Kaempferol	Urine	N	U	Y	U	U	U	Y	U
	Dimethylamine	Urine	N	U	U	U	U	U	Y	U
	Glutamine	Urine	N	U	U	U	U	U	Y	U
	3-Methylhistidine	Urine	N	U	U	U	U	Y	Y	U
	Trigonelline	Urine	N	Y	U	U	U	U	N	U
		Plasma/serum	N	U	U	N	U	U	Y	U
	Pipecolic acid	Plasma/serum	N	U	U	Y	U	U	Y	U
	Indolepropionate	Plasma/serum	U	U	U	N	U	U	U	U
	S-Methylcysteine	Plasma/serum	U	U	U	Y	U	U	U	U
	*N*-Acetyl-ornithine	Plasma/serum	U	U	U	N	U	U	U	U

Possible answers are Y (yes), N (No), or U (unknown or uncertain)

Questions1. Is the marker compound plausible as a specific biomarker of food intake for the food or food group (chemical/biological plausibility)?2. Is there a dose-response relationship at relevant intake levels of the targeted food (quantitative aspect)?3. Is the biomarker kinetics described adequately to make a wise choice of sample type, frequency and time window (time-response)?4. Has the marker been shown to be robust after intake of complex meals reflecting dietary habits of the targeted population (robustness)?5. Has the marker been shown to compare well with other markers or questionnaire data for the same food/food group (reliability)?6. Is the marker chemically and biologically stable during biospecimen collection and storage, making measurements reliable and feasible (stability)?7. Are analytical variability (CV%), accuracy, sensitivity and specificity known as adequate for at least one reported analytical method (analytical performance)?8. Has the analysis been successfully reproduced in another laboratory (reproducibility)?

Regarding isoflavone metabolites *O*-DMA, dihydrogenistein, and dihydrodaidzein, the urinary excretion of these compounds was weakly associated with soy food intake [104], and furthermore, these metabolized isoflavones are also reported to be present in human urine following red clover supplementation [105]. Taken together, this suggests the non-specificity of these metabolites after soy intake.

From the studies examined, it is clear that genistein and daidzein represent biomarkers of soy intake in different population groups. The influence of gender on soy isoflavone excretion was reported with urinary genistein recovery found to be higher after consumption of soy-based foods in women, while no differences were observed in males. Urinary daidzein recovery was not affected by gender, but conversion to metabolite equol seems to be influenced by chemical composition of the isoflavones ingested and the dietary factors such as fiber and carbohydrate [106]. Gender differences were also reported with longer half-lives for plasma genistein and daidzein in females as compared to males [79]. Furthermore, another factor that needs to be considered is the effect of interindividual variation. Numerous studies have shown considerable interindividual variation between participants in the plasma and urinary concentrations of isoflavones [19, 79, 107] and their metabolites especially equol demonstrating multifold interindividual variation [51, 80, 107]. It might be caused due to differences in absorption and metabolism, differential gut microflora composition, genetic variation in transporter genes, dietary fat, carbohydrate, and fiber intake [41, 51, 108–110].

The lignan phytoestrogens enterodiol, enterolactone, and matairesinol are widely distributed in many plant classes, and their presence is more prevalent in whole grains and fiber-containing plant foods (wheat, oats, rye), oilseeds (flax seeds and sesame seeds), and various other fruits and vegetables [70, 93, 111]. According to a study conducted at USDA [112], high concentrations of lignans were reported for flaxseeds (28,800–94,500 μg/100 g), cereal grains (168.1–1084.1 μg/100 g), vegetables (389.1–6344 μg/100 g), and fruits (229–2354 μg/100 g), while lower levels were reported for soy (130–1268 μg/100 g). The urinary excretion levels of lignans as reported in the lignin phytoestrogen section is high after consumption of sources other than soy-based foods suggesting the limited role of soy as a specific source of lignan phytoestrogens.

In parallel, an additional search was conducted for compounds identified following pulse consumption to examine the candidate biomarkers for specificity for pulses. The assessment of data presented in Table 2 revealed that kaempferol is present in a wide range of edible plants such as tea, broccoli, cabbage, kale, leek, tomato, strawberries, and grapes [113]. Consequently, kaempferol has been detected in biological samples after intake of other foods such as fruits and vegetables [114, 115], onions [116, 117], tea [116, 118–121], other phenol-rich foods [122], and other food sources [123–125]. Dimethylamine has been proposed as a marker of fish consumption [126], while 3-methylhistidine is a marker of chicken intake [127]. On the other hand, trigonelline has also been proposed as a biomarker of coffee with high concentrations reported after coffee intake [128–131]. Regarding pipecolic acid, it can be found in the urine or serum after consumption of black soybean peptide [132] and whole grain-enriched diet [133]; and indole propionate was reported as marker of red meat and eggs intake [134]. On the other hand, no relevant papers were found to evaluate the specificity of *S*-methylcysteine and *N*-acetyl-ornithine. Based on the above information, the compounds detected in the biofluids after consumption of beans are also present in other food sources, and hence, none of these compounds could be considered as specific biomarkers of bean intake when evaluated alone.

## Conclusions

Although many compounds have been suggested as biomarkers for soy, pulses, and legumes in general, the validation of these compounds against other markers for the same food/food group needs to be performed. Overall, genistein and daidzein could potentially be considered as relevant markers of soy considering various evidence from the literature such as dose-response relationships and the suitability for detecting both acute and habitual intake as evidenced from intervention and cross-sectional studies. In addition, both genistein and daidzein were proven as good estimates of soy intake as evidenced from long-term exposure studies further marking their status as validated biomarkers. Due to the dearth of information on biomarkers of pulses, further discovery and validation studies are needed in this area in order to identify reliable biomarkers of pulse intake.

## Additional file

Additional file 1: **Table S1.** Keywords related to the name and synonyms of the each potential biomarker of soy/pulses intake used in the search strategy. (DOCX 17 kb)

## Abbreviations

FFQ: Food frequency questionnaire
*O*-DMA: *O*-Desmethylangolensin
SFQ: Soy food questionnaire

## References

[1] Maphosa Y, Jideani VA. The role of legumes in human nutrition. Functional Food Maria Chavarri, IntechOpen; 2017. 10.5772/intechopen69127. Available from: https://www.intechopen.com/books/functional-food-improve-health-through-adequate-food/the-role-of-legumes-in-human-nutrition.

[2] Bouchenak M, Lamri-Senhadji M (2013). Nutritional quality of legumes, and their role in cardiometabolic risk prevention: a review. J Med Food.

[3] Messina M (2016). Soy and health update: evaluation of the clinical and epidemiologic literature. Nutrients.

[4] Polak R, Phillips EM, Campbell A (2015). Legumes: health benefits and culinary approaches to increase intake. Clin Diabetes.

[5] Messina MJ (1999). Legumes and soybeans: overview of their nutritional profiles and health effects. Am J Clin Nutr.

[6] Bhagwat S, Haytowitz DB, Holden JM (2008). USDA database for the isoflavone content of selected foods release 2.0. Nutrient Data Laboratory, Beltsville Human Nutrition Research Center, Agricultural Research Service U.S. Department of Agriculture.

[7] Liggins J, Bluck LJ, Runswick S, Atkinson C, Coward WA, Bingham SA (2000). Daidzein and genistein contents of vegetables. Br J Nutr.

[8] Bazzano LA, Thompson AM, Tees MT, Nguyen CH, Winham DM (2011). Non-soy legume consumption lowers cholesterol levels: a meta-analysis of randomized controlled trials. Nutr Metab Cardiovasc Dis.

[9] Ley SH, Hamdy O, Mohan V, Hu FB (2014). Prevention and management of type 2 diabetes: dietary components and nutritional strategies. Lancet.

[10] Heber D (2008). Plant foods and phytochemicals in human health.

[11] Barnes S, Kirk M, Coward L (1994). Isoflavones and their conjugates in soy foods: extraction conditions and analysis by HPLC-mass spectrometry. J Agric Food Chem.

[12] Adlercreutz CH, Goldin BR, Gorbach SL, Hockerstedt KA, Watanabe S, Hamalainen EK (1995). Soybean phytoestrogen intake and cancer risk. J Nutr.

[13] Setchell KD, Borriello SP, Hulme P, Kirk DN, Axelson M (1984). Nonsteroidal estrogens of dietary origin: possible roles in hormone-dependent disease. Am J Clin Nutr.

[14] Dong JY, Qin LQ (2011). Soy isoflavones consumption and risk of breast cancer incidence or recurrence: a meta-analysis of prospective studies. Breast Cancer Res Treat.

[15] Birru RL, Ahuja V, Vishnu A, Evans RW, Miyamoto Y, Miura K (2016). The impact of equol-producing status in modifying the effect of soya isoflavones on risk factors for CHD: a systematic review of randomised controlled trials. J Nutr Sci.

[16] Goldwyn S, Lazinsky A, Wei H (2000). Promotion of health by soy isoflavones: efficacy, benefit and safety concerns. Drug Metabol Drug Interact.

[17] Adlercreutz H (1998). Epidemiology of phytoestrogens. Bailliere Clin Endocrinol Metab.

[18] Wiseman H (2000). The therapeutic potential of phytoestrogens. Expert Opin Investig Drugs.

[19] Xu X, Wang HJ, Murphy PA, Cook L, Hendrich S (1994). Daidzein is a more bioavailable soymilk isoflavone than is genistein in adult women. J Nutr.

[20] Hsieh CY, Santell RC, Haslam SZ, Helferich WG (1998). Estrogenic effects of genistein on the growth of estrogen receptor-positive human breast cancer (MCF-7) cells *in vitro* and *in vivo*. Cancer Res.

[21] Ju YH, Fultz J, Allred KF, Doerge DR, Helferich WG (2006). Effects of dietary daidzein and its metabolite, equol, at physiological concentrations on the growth of estrogen-dependent human breast cancer (MCF-7) tumors implanted in ovariectomized athymic mice. Carcinogenesis.

[22] Ziaei S, Halaby R (2017). Dietary Isoflavones and Breast Cancer Risk. Medicines (Basel).

[23] Nothlings U, Schulze MB, Weikert C, Boeing H, van der Schouw YT, Bamia C (2008). Intake of vegetables, legumes, and fruit, and risk for all-cause, cardiovascular, and cancer mortality in a European diabetic population. J Nutr.

[24] Jayalath VH, de Souza RJ, Sievenpiper JL, Ha V, Chiavaroli L, Mirrahimi A (2014). Effect of dietary pulses on blood pressure: a systematic review and meta-analysis of controlled feeding trials. Am J Hypertens.

[25] Kim SJ, de Souza RJ, Choo VL, Ha V, Cozma AI, Chiavaroli L (2016). Effects of dietary pulse consumption on body weight: a systematic review and meta-analysis of randomized controlled trials. Am J Clin Nutr.

[26] Li SS, Kendall CW, de Souza RJ, Jayalath VH, Cozma AI, Ha V (2014). Dietary pulses, satiety and food intake: a systematic review and meta-analysis of acute feeding trials. Obesity.

[27] Ha V, Sievenpiper JL, de Souza RJ, Jayalath VH, Mirrahimi A, Agarwal A (2014). Effect of dietary pulse intake on established therapeutic lipid targets for cardiovascular risk reduction: a systematic review and meta-analysis of randomized controlled trials. Can Med Assoc J.

[28] Sievenpiper JL, Kendall CW, Esfahani A, Wong JM, Carleton AJ, Jiang HY (2009). Effect of non-oil-seed pulses on glycaemic control: a systematic review and meta-analysis of randomised controlled experimental trials in people with and without diabetes. Diabetologia.

[29] Bingham SA (2002). Biomarkers in nutritional epidemiology. Public Health Nutr.

[30] Kipnis V, Midthune D, Freedman L, Bingham S, Day NE, Riboli E (2002). Bias in dietary-report instruments and its implications for nutritional epidemiology. Public Health Nutr.

[31] Kuhnle GG (2012). Nutritional biomarkers for objective dietary assessment. J Sci Food Agric.

[32] O'Gorman A, Gibbons H, Brennan L (2013). Metabolomics in the identification of biomarkers of dietary intake. Comput Struct Biotechnol J.

[33] Tucker KL, Smith CE, Lai CQ, Ordovas JM (2013). Quantifying diet for nutrigenomic studies. Annu Rev Nutr.

[34] Crews H, Alink G, Andersen R, Braesco V, Holst B, Maiani G (2001). A critical assessment of some biomarker approaches linked with dietary intake. Br J Nutr.

[35] Moher D, Liberati A, Tetzlaff J, Altman DG, Group P (2009). Preferred reporting items for systematic reviews and meta-analyses: the PRISMA statement. PLoS Med.

[36] Pratico G, Gao Q, Scalbert A, Vergeres G, Kolehmainen M, Manach C (2018). Guidelines for Biomarker of Food Intake Reviews (BFIRev): how to conduct an extensive literature search for biomarker of food intake discovery. Genes Nutr.

[37] Dragsted LO, Gao Q, Scalbert A, Vergeres G, Kolehmainen M, Manach C (2018). Validation of biomarkers of food intake-critical assessment of candidate biomarkers. Genes Nutr.

[38] Seow A, Shi CY, Franke AA, Hankin JH, Lee HP, Yu MC (1998). Isoflavonoid levels in spot urine are associated with frequency of dietary soy intake in a population-based sample of middle-aged and older Chinese in Singapore. Cancer Epidemiol Biomark Prev.

[39] Chen Z, Zheng W, Custer LJ, Dai Q, Shu XO, Jin F (1999). Usual dietary consumption of soy foods and its correlation with the excretion rate of isoflavonoids in overnight urine samples among Chinese women in Shanghai. Nutr Cancer.

[40] Jaceldo-Siegl K, Fraser GE, Chan J, Franke A, Sabate J (2008). Validation of soy protein estimates from a food-frequency questionnaire with repeated 24-h recalls and isoflavonoid excretion in overnight urine in a Western population with a wide range of soy intakes. Am J Clin Nutr.

[41] Lampe JW, Gustafson DR, Hutchins AM, Martini MC, Li S, Wahala K (1999). Urinary isoflavonoid and lignan excretion on a Western diet: relation to soy, vegetable, and fruit intake. Cancer Epidem Biomar.

[42] Atkinson C, Skor HE, Fitzgibbons ED, Scholes D, Chen C, Wahala K (2002). Overnight urinary isoflavone excretion in a population of women living in the United States, and its relationship to isoflavone intake. Cancer Epidem Biomar.

[43] Tseng M, Olufade T, Kurzer MS, Wahala K, Fang CY, van der Schouw YT (2008). Food frequency questionnaires and overnight urines are valid indicators of daidzein and genistein intake in U.S. women relative to multiple 24-h urine samples. Nutr Cancer.

[44] Maskarinec G, Singh S, Meng LX, Franke AA (1998). Dietary soy intake and urinary isoflavone excretion among women from a multiethnic population. Cancer Epidem Biomar.

[45] Verkasalo PK, Appleby PN, Allen NE, Davey G, Adlercreutz H, Key TJ (2001). Soya intake and plasma concentrations of daidzein and genistein: validity of dietary assessment among eighty British women (Oxford arm of the European Prospective Investigation into Cancer and Nutrition). Brit J Nutr.

[46] Frankenfeld CL, Patterson RE, Kalhorn TF, Skor HE, Howald WN, Lampe JW (2002). Validation of a soy food frequency questionnaire with plasma concentrations of isoflavones in US adults. J Am Diet Assoc.

[47] Frankenfeld CL, Patterson RE, Horner NK, Neuhouser ML, Skor HE, Kalhorn TF (2003). Validation of a soy food-frequency questionnaire and evaluation of correlates of plasma isoflavone concentrations in postmenopausal women. Am J Clin Nutr.

[48] Wu AH, Yu MC, Tseng CC, Twaddle NC, Doerge DR (2004). Plasma isoflavone levels versus self-reported soy isoflavone levels in Asian-American women in Los Angeles County. Carcinogenesis.

[49] Frankenfeld CL, Lampe JW, Shannon J, Gao DL, Ray RM, Prunty J (2004). Frequency of soy food consumption and serum isoflavone concentrations among Chinese women in Shanghai. Public Health Nutr.

[50] Kelly GE, Nelson C, Waring MA, Joannou GE, Reeder AY (1993). Metabolites of dietary (soya) isoflavones in human urine. Clin Chim Acta.

[51] Rowland IR, Wiseman H, Sanders TAB, Adlercreutz H, Bowey EA (2000). Interindividual variation in metabolism of soy isoflavones and lignans: influence of habitual diet on equol production by the gut microflora. Nutr Cancer.

[52] Kirkman LM, Lampe JW, Campbell DR, Martini MC, Slavin JL (1995). Urinary lignan and isoflavonoid excretion in men and women consuming vegetable and soy diets. Nutr Cancer.

[53] Karr SC, Lampe JW, Hutchins AM, Slavin JL (1997). Urinary isoflavonoid excretion in humans is dose dependent at low to moderate levels of soy-protein consumption. Am J Clin Nutr.

[54] Gooderham MJ, Adlercreutz H, Ojala ST, Wahala K, Holub BJ (1996). A soy protein isolate rich in genistein and daidzein and its effects on plasma isoflavone concentrations, platelet aggregation, blood lipids and fatty acid composition of plasma phospholipid in normal men. J Nutr.

[55] Cassidy A, Bingham S, Setchell KDR (1994). Biological effects of a diet of soy protein-rich in isoflavones on the menstrual-cycle of premenopausal women. Am J Clin Nutr.

[56] Hutchins AM, Slavin JL, Lampe JW (1995). Urinary isoflavonoid phytoestrogen and lignan excretion after consumption of fermented and unfermented soy products. J Am Diet Assoc.

[57] Klejdus B, Vacek J, Benesova L, Kopecky J, Lapcik O, Kuban V (2007). Rapid-resolution HPLC with spectrometric detection for the determination and identification of isoflavones in soy preparations and plant extracts. Anal Bioanal Chem.

[58] Franke AA, Hebshi SM, Pagano I, Kono N, Mack WJ, Hodis HN (2010). Urine accurately reflects circulating isoflavonoids and ascertains compliance during soy intervention. Cancer Epidem Biomar.

[59] Kano M, Takayanagi T, Harada K, Sawada S, Ishikawa F (2006). Bioavailability of isoflavones after ingestion of soy beverages in healthy adults. J Nutr.

[60] Shinkaruk S, Durand M, Lamothe V, Carpaye A, Martinet A, Chantre P (2012). Bioavailability of glycitein relatively to other soy isoflavones in healthy young Caucasian men. Food Chem.

[61] Maskarinec G, Watts K, Kagihara J, Hebshi SM, Franke AA (2008). Urinary isoflavonoid excretion is similar after consuming soya milk and miso soup in Japanese-American women. Brit J Nutr.

[62] Izumi T, Piskula MK, Osawa S, Obata A, Tobe K, Saito M (2000). Soy isoflavone aglycones are absorbed faster and in higher amounts than their glucosides in humans. J Nutr.

[63] Rufer CE, Bub A, Moseneder J, Winterhalter P, Sturtz M, Kulling SE (2008). Pharmacokinetics of the soybean isoflavone daidzein in its aglycone and glucoside form: a randomized, double-blind, crossover study. Am J Clin Nutr.

[64] Setchell KD, Brown NM, Desai P, Zimmer-Nechemias L, Wolfe BE, Brashear WT (2001). Bioavailability of pure isoflavones in healthy humans and analysis of commercial soy isoflavone supplements. J Nutr.

[65] Richelle M, Pridmore-Merten S, Bodenstab S, Enslen M, Offord EA (2002). Hydrolysis of isoflavone glycosides to aglycones by beta-glycosidase does not alter plasma and urine isoflavone pharmacokinetics in postmenopausal women. J Nutr.

[66] Zubik L, Meydani M (2003). Bioavailability of soybean isoflavones from aglycone and glucoside forms in American women. Am J Clin Nutr.

[67] King RA, Bursill DB (1998). Plasma and urinary kinetics of the isoflavones daidzein and genistein after a single soy meal in humans. Am J Clin Nutr.

[68] Setchell KDR, Brown NM, Desai PB, Zimmer-Nechimias L, Wolfe B, Jakate AS (2003). Bioavailability, disposition, and dose-response effects of soy isoflavones when consumed by healthy women at physiologically typical dietary intakes. J Nutr.

[69] Anupongsanugool E, Teekachunhatean S, Rojanasthien N, Pongsatha S, Sangdee C (2005). Pharmacokinetics of isoflavones, daidzein and genistein, after ingestion of soy beverage compared with soy extract capsules in postmenopausal Thai women. BMC Clin Pharmacol.

[70] Lampe JW (2003). Isoflavonoid and lignan phytoestrogens as dietary biomarkers. J Nutr.

[71] Mathey J, Lamothe V, Coxam V, Potier M, Sauvant P, Bennetau-Pelissero C (2006). Concentrations of isoflavones in plasma and urine of post-menopausal women chronically ingesting high quantities of soy isoflavones. J Pharm Biomed Anal.

[72] Maskarinec G, Oshiro C, Morimoto Y, Hebshi S, Novotny R, Franke AA (2005). Urinary isoflavone excretion as a compliance measure in a soy intervention among young girls: a pilot study. Eur J Clin Nutr.

[73] Morimoto Y, Beckford F, Franke AA, Maskarinec G (2014). Urinary isoflavonoid excretion as a biomarker of dietary soy intake during two randomized soy trials. Asia Pac J Clin Nutr.

[74] Genovese MI, Lopes Barbosa AC, Pinto MD, Lajolo FM (2007). Commercial soy protein ingredients as isoflavone sources for functional foods. Plant Food Hum Nutr.

[75] Wang C, Ma Q, Pagadala S, Sherrard MS, Krishnan PG (1998). Changes of isoflavones during processing of soy protein isolates. J Am Oil Chem Soc.

[76] Allred CD, Twaddle NC, Allred KF, Goeppinger TS, Churchwell MI, Ju YH (2005). Soy processing affects metabolism and disposition of dietary isoflavones in ovariectomized balb/c mice. J Agric Food Chem.

[77] Andrade JE, Twaddle NC, Helferich WG, Doerge DR (2010). Absolute bioavailability of isoflavones from soy protein isolate-containing food in female balb/c mice. J Agric Food Chem.

[78] Vergne S, Titier K, Bernard V, Asselineau J, Durand M, Lamothe V (2007). Bioavailability and urinary excretion of isoflavones in humans: effects of soy-based supplements formulation and equol production. J Pharmaceut Biomed.

[79] Lu LJW, Lin SN, Grady JJ, Nagamani M, Anderson KE (1996). Altered kinetics and extent of urinary daidzein and genistein excretion in women during chronic soya exposure. Nutr Cancer.

[80] Lampe JW, Karr SC, Hutchins AM, Slavin JL (1998). Urinary equol excretion with a soy challenge: influence of habitual diet. P Soc Exp Biol Med.

[81] Maskarinec G, Yamakawa R, Hebshi S, Franke AA (2007). Urinary isoflavonoid excretion and soy consumption in three generations of Japanese women in Hawaii. Eur J Clin Nutr.

[82] Saunier K, Dore J (2002). Gastrointestinal tract and the elderly: functional foods, gut microflora and healthy ageing. Dig Liver Dis.

[83] Van Tongeren SP, Slaets JPJ, Harmsen HJM, Welling GW (2005). Fecal microbiota composition and frailty. Appl Environ Microbiol.

[84] Franke AA, Lai JF, Halm BM, Pagano I, Kono N, Mack WJ (2012). Equol production changes over time in postmenopausal women. J Nutr Biochem.

[85] Frankenfeld CL, Atkinson C, Thomas WK, Gonzalez A, Jokela T, Wahala K (2005). High concordance of daidzein-metabolizing phenotypes in individuals measured 1 to 3 years apart. Brit J Nutr.

[86] Nettleton JA, Greany KA, Thomas W, Wangen KE, Adlercreutz H, Kurzer MS (2004). Plasma phytoestrogens are not altered by probiotic consumption in postmenopausal women with and without a history of breast cancer. J Nutr.

[87] Setchell KDR, Brown NM, Summer S, King EC, Heubi JE, Cole S (2013). Dietary factors influence production of the soy isoflavone metabolite S-(−) equol in healthy adults. J Nutr.

[88] Frankenfeld CL (2011). O-Desmethylangolensin: the importance of equol’s lesser known cousin to human health. Adv Nutr.

[89] Watanabe S, Yamaguchi M, Sobue T, Takahashi T, Miura T, Arai Y (1998). Pharmacokinetics of soybean isoflavones in plasma, urine and feces of men after ingestion of 60 g baked soybean powder (kinako). J Nutr.

[90] Franke AA, Morimoto Y, Yeh LM, Maskarinec G (2006). Urinary isoflavonoids as a dietary compliance measure among premenopausal women. Asia Pac J Clin Nutr.

[91] Heinonen S, Wahala K, Adlercreutz H (1999). Identification of isoflavone metabolites dihydrodaidzein, dihydrogenistein, 6′-OH-O-dma, and cis-4-OH-equol in human urine by gas chromatography-mass spectroscopy using authentic reference compounds. Anal Biochem.

[92] Zhang Y, Hendrich S, Murphy PA (2003). Glucuronides are the main isoflavone metabolites in women. J Nutr.

[93] Adlercreutz H, Honjo H, Higashi A, Fotsis T, Hamalainen E, Hasegawa T (1991). Urinary-excretion of lignans and isoflavonoid phytoestrogens in Japanese men and women consuming a traditional Japanese diet. Am J Clin Nutr.

[94] Perera T, Young MR, Zhang ZY, Murphy G, Colburn NH, Lanza E (2015). Identification and monitoring of metabolite markers of dry bean consumption in parallel human and mouse studies. Mol Nutr Food Res.

[95] Bonetti A, Marotti I, Dinelli G (2007). Urinary excretion of kaempferol from common beans (Phaseolus vulgaris L.) in humans. Int J Food Sci Nutr.

[96] Andersen MBS, Kristensen M, Manach C, Pujos-Guillot E, Poulsen SK, Larsen TM (2014). Discovery and validation of urinary exposure markers for different plant foods by untargeted metabolomics. Anal Bioanal Chem.

[97] Madrid-Gambin F, Llorach R, Vazquez-Fresno R, Urpi-Sarda M, Almanza-Aguilera E, Garcia-Aloy M (2017). Urinary (1) H nuclear magnetic resonance metabolomic fingerprinting reveals biomarkers of pulse consumption related to energy-metabolism modulation in a subcohort from the PREDIMED study. J Proteome Res.

[98] Posma JM, Garcia-Perez I, Heaton JC, Burdisso P, Mathers JC, Draper J (2017). Integrated analytical and statistical two-dimensional spectroscopy strategy for metabolite identification: application to dietary biomarkers. Anal Chem.

[99] Kaufman PB, Duke JA, Brielmann H, Boik J, Hoyt JE (1997). A comparative survey of leguminous plants as sources of the isoflavones, genistein and daidzein: implications for human nutrition and health. J Altern Complement Med.

[100] Tsunoda N, Pomeroy S, Nestel P (2002). Absorption in humans of isoflavones from soy and red clover is similar. J Nutr.

[101] Busby MG, Jeffcoat AR, Bloedon LT, Koch MA, Black T, Dix KJ (2002). Clinical characteristics and pharmacokinetics of purified soy isoflavones: single-dose administration to healthy men. Am J Clin Nutr.

[102] Wilkinson AP, Wahala K, Williamson G (2002). Identification and quantification of polyphenol phytoestrogens in foods and human biological fluids. J Chromatogr B Anal Technol Biomed Life Sci.

[103] Ogita T, Watanabe J, Wakagi M, Nakamichi K, Komiyama S, Takebayashi J (2015). Evaluation of a method to quantify isoflavones in soybean by single and multi-laboratory validation studies. Food Sci Technol Res.

[104] Wu X, Cai H, Gao YT, Dai Q, Li H, Cai Q (2012). Correlations of urinary phytoestrogen excretion with lifestyle factors and dietary intakes among middle-aged and elderly Chinese women. Int J Mol Epidemiol Genetics.

[105] Lipovac M, Pfitscher A, Hobiger S, Laschitz T, Imhof M, Chedraui P (2015). Red clover isoflavone metabolite bioavailability is decreased after fructooligosaccharide supplementation. Fitoterapia.

[106] Faughnan MS, Hawdon A, Ah-Singh E, Brown J, Millward DJ, Cassidy A (2004). Urinary isoflavone kinetics: the effect of age, gender, food matrix and chemical composition. Br J Nutr.

[107] Kelly GE, Joannou GE, Reeder AY, Nelson C, Waring MA (1995). The variable metabolic response to dietary isoflavones in humans. Proc Soc Exp Biol Med.

[108] Nielsen IL, Williamson G (2007). Review of the factors affecting bioavailability of soy isoflavones in humans. Nutr Cancer.

[109] Wakeling LA, Ford D (2012). Polymorphisms in genes involved in the metabolism and transport of soy isoflavones affect the urinary metabolite profile in premenopausal women following consumption of a commercial soy supplement as a single bolus dose. Mol Nutr Food Res.

[110] Van der Velpen V, Hollman PC, van Nielen M, Schouten EG, Mensink M, Van't Veer P (2014). Large inter-individual variation in isoflavone plasma concentration limits use of isoflavone intake data for risk assessment. Eur J Clin Nutr.

[111] Milder IEJ, Arts ICW, van de Putte B, Venema DP, Hollman PCH (2005). Lignan contents of Dutch plant foods: a database including lariciresinol, pinoresinol, secoisolariciresinol and matairesinol. Brit J Nutr.

[112] Meagher LP, Beecher GR (2000). Assessment of data on the lignan content of foods. J Food Compos Anal.

[113] Calderon-Montano JM, Burgos-Moron E, Perez-Guerrero C, Lopez-Lazaro M (2011). A review on the dietary flavonoid kaempferol. Mini-Rev Med Chem.

[114] Brevik A, Rasmussen SE, Drevon CA, Andersen LF (2004). Urinary excretion of flavonoids reflects even small changes in the dietary intake of fruits and vegetables. Cancer Epidemiol Biomark Prev.

[115] Mennen LI, Sapinho D, Ito H, Bertrais S, Galan P, Hercberg S (2006). Urinary flavonoids and phenolic acids as biomarkers of intake for polyphenol-rich foods. Brit J Nutr.

[116] de Vries JHM, Hollman PCH, Meyboom S, Buysman MNCP, Zock PL, Van Staveren WA (1998). Plasma concentrations and urinary excretion of the antioxidant flavonols quercetin and kaempferol as biomarkers for dietary intake. Am J Clin Nutr.

[117] Hong Y, Mitchell AE (2004). Metabolic profiling of flavonol metabolites in human urine by liquid chromatography and tandem mass spectrometry. J Agric Food Chem.

[118] Brantsaeter AL, Haugen M, Rasmussen SE, Alexander J, Samuelsen SO, Meltzer HM (2007). Urine flavonoids and plasma carotenoids in the validation of fruit, vegetable and tea intake during pregnancy in the Norwegian Mother and Child Cohort Study (MoBa). Public Health Nutr.

[119] Hollman PC, Van Het Hof KH, Tijburg LB, Katan MB (2001). Addition of milk does not affect the absorption of flavonols from tea in man. Free Radic Res.

[120] Krogholm KS, Bysted A, Brantsaeter AL, Jakobsen J, Rasmussen SE, Kristoffersen L (2012). Evaluation of flavonoids and enterolactone in overnight urine as intake biomarkers of fruits, vegetables and beverages in the Inter99 cohort study using the method of triads. Brit J Nutr.

[121] Nielsen SE, Freese R, Kleemola P, Mutanen M (2002). Flavonoids in human urine as biomarkers for intake of fruits and vegetables. Cancer Epidem Biomar.

[122] Kim HY, Kim OH, Sung MK (2003). Effects of phenol-depleted and phenol-rich diets on blood markers of oxidative stress, and urinary excretion of quercetin and kaempferol in healthy volunteers. J Am Coll Nutr.

[123] DuPont MS, Day AJ, Bennett RN, Mellon FA, Kroon PA (2004). Absorption of kaempferol from endive, a source of kaempferol-3-glucuronide, in humans. Eur J Clin Nutr.

[124] Grinder-Pedersen L, Rasmussen SE, Bugel S, Jorgensen LV, Dragsted LO, Gundersen V (2003). Effect of diets based on foods from conventional versus organic production on intake and excretion of flavonoids and markers of antioxidative defense in humans. J Agric Food Chem.

[125] Wang FM, Yao TW, Zeng S (2003). Disposition of quercetin and kaempferol in human following an oral administration of Ginkgo biloba extract tablets. Eur J Drug Metab Pharmacokinet.

[126] Tsikas D, Thum T, Becker T, Pham VV, Chobanyan K, Mitschke A (2007). Accurate quantification of dimethylamine (DMA) in human urine by gas chromatography-mass spectrometry as pentafluorobenzamide derivative: evaluation of the relationship between DMA and its precursor asymmetric dimethylarginine (ADMA) in health and disease. J Chromatogr B Anal Technol Biomed Life Sci.

[127] Huszar G, Golenwsky G, Maiocco J, Davis E (1983). Urinary 3-methylhistidine excretion in man: the role of protein-bound and soluble 3-methylhistidine. Br J Nutr.

[128] Guertin KA, Loftfield E, Boca SM, Sampson JN, Moore SC, Xiao Q (2015). Serum biomarkers of habitual coffee consumption may provide insight into the mechanism underlying the association between coffee consumption and colorectal cancer. Am J Clin Nutr.

[129] Lang R, Wahl A, Stark T, Hofmann T (2011). Urinary N-methylpyridinium and trigonelline as candidate dietary biomarkers of coffee consumption. Mol Nutr Food Res.

[130] Rothwell JA, Fillatre Y, Martin JF, Lyan B, Pujos-Guillot E, Fezeu L (2014). New biomarkers of coffee consumption identified by the non-targeted metabolomic profiling of cohort study subjects. PLoS One.

[131] Madrid-Gambin F, Garcia-Aloy M, Vazquez-Fresno R, Vegas-Lozano E, Jubany MCRD, Misawa K (2016). Impact of chlorogenic acids from coffee on urine metabolome in healthy human subjects. Food Res Int.

[132] Kim MJ, Yang HJ, Kim JH, Ahn CW, Lee JH, Kim KS (2013). Obesity-related metabolomic analysis of human subjects in black soybean peptide intervention study by ultraperformance liquid chromatography and quadrupole-time-of-flight mass spectrometry. J Obes.

[133] Hanhineva K, Lankinen MA, Pedret A, Schwab U, Kolehmainen M, Paananen J (2015). Nontargeted metabolite profiling discriminates diet-specific biomarkers for consumption of whole grains, fatty fish, and bilberries in a randomized controlled trial. J Nutr.

[134] Guertin KA, Moore SC, Sampson JN, Huang WY, Xiao Q, Stolzenberg-Solomon RZ (2014). Metabolomics in nutritional epidemiology: identifying metabolites associated with diet and quantifying their potential to uncover diet-disease relations in populations. Am J Clin Nutr.

