# Correction to: Biomarkers of legume intake in human intervention and observational studies: a systematic review

**DOI:** 10.1186/s12263-018-0616-4

**Published:** 2018-10-16

**Authors:** Pedapati S C Sri Harsha, Roshaida Abdul Wahab, Mar Garcia-Aloy, Francisco Madrid-Gambin, Sheila Estruel-Amades, Bernhard Watzl, Cristina Andrés-Lacueva, Lorraine Brennan

**Affiliations:** 1 UCD School of Agriculture and Food Science, UCD Institute of Food and Health, UCD, Belfield, Dublin 4, Ireland; 2 Biomarkers and Nutrimetabolomic Laboratory, Department of Nutrition, Food Sciences and Gastronomy, XaRTA, INSA, Faculty of Pharmacy and Food Sciences, University of Barcelona, Barcelona, Spain; 3 CIBER de Fragilidad y Envejecimiento Saludable (CIBERFES), Instituto de Salud Carlos III, Barcelona, Spain; 4 Department of Physiology and Biochemistry of Nutrition, Max Rubner-Institut, Federal Research Institute of Nutrition and Food, Karlsruhe, Germany

## Correction

Following publication of the original article [1], the authors reported an error with the third author’s name, whereby the given name was ‘Mar Garcia’ and the family name ‘Aloy’, while the given name is in fact ‘Mar’ and the family name ‘Garcia-Aloy’.

As such, the original article [1] has been updated accordingly.
