# Supplementary material for: Biomarkers of legume intake in human intervention and observational studies: a systematic review
Source: Genes Nutr. 2018 Sep 10;13:25. doi: 10.1186/s12263-018-0614-6 (PMC6131749; doi:10.1186/s12263-018-0614-6)
Supplement: Supplementary file 1 — Table S1. Keywords related to the name and synonyms of the each potential biomarker of soy/pulses intake used in the search strategy. (DOCX 17 kb) [file 12263_2018_614_MOESM1_ESM.docx]

**Additional file 1: Table S1.** Keywords related to the name and synonyms of the each potential biomarker of soy/pulses intake used in the search strategy

| **Compound** | **Keywords** |
| --- | --- |
| Genistein | Genistein OR 4',5,7-Trihydroxyisoflavone OR 4',5,7-Trihydroxy-Isoflavone OR 4,5,7-Trihydroxy Iso-Flavone OR 5,7,4'-Trihydroxyisoflavone OR 5,7-Dihydroxy-3-(4-hydroxyphenyl)-4H-1-Benzopyran-4-one OR Genisteol OR Genisterin OR Prunetol OR Sophoricol OR 5,7-dihydroxy-3-(4-hydroxyphenyl)-4H-chromen-4-one |
| Dihydrogenistein | Dihydrogenistein OR 4',5,7-Trihydroxy-Isoflavanone OR 4',5,7-Trihydroxyisoflavan-4-one OR 5,7-dihydroxy-3-(4-hydroxyphenyl)-3,4-dihydro-2H-1-benzopyran-4-one |
| Dihydrodaidzein | Dihydrodaidzein OR 2,3-Dihydro-7-hydroxy-3-(4-hydroxyphenyl)-4H-1-Benzopyran-4-one OR 4',7-Dihydroxy-Isoflavanone OR 7-Hydroxy-3-(4-hydroxyphenyl)chroman-4-one OR 7-hydroxy-3-(4-hydroxyphenyl)-3,4-dihydro-2H-1-benzopyran-4-one |
| Enterodiol | Enterodiol OR (2R,3R)-2,3-bis[(3-hydroxyphenyl)methyl]-1,4-Butanediol OR [R-(R*,R*)]-2,3-bis[(3-hydroxyphenyl)methyl]-1,4-Butanediol OR (2R,3R)-2,3-bis[(3-hydroxyphenyl)methyl]butane-1,4-diol |
| Enterolactone | Enterolactone OR 2,3-Bis(3'-hydroxybenzyl)butyrolactone OR 3,4-Bis((3-hydroxyphenyl)methyl)dihydro-2-(3H)-furanone OR Dihydro-3,4-bis((3-hydroxyphenyl)methyl)-2(3H)-Furanone OR 3,4-bis[(3-hydroxyphenyl)methyl]oxolan-2-one |
| Matairesinol | Matairesinol OR 4,4'-Dihydroxy-3,3'-dimethoxylignan-9,9'-olide OR Dihydro-3,4-bis[(4-hydroxy-3-methoxyphenyl)methyl]-2(3H)-furanone OR Dihydro-3,4-divanillyl-2(3H)-furanone OR 3,4-bis[(4-hydroxy-3-methoxyphenyl)methyl]oxolan-2-one |
| Daidzein | Daidzein OR 4',7-Dihydroxy-Isoflavone OR 4',7-Dihydroxyisoflavone OR 7,4'-Dihydroxyisoflavone OR 7-Hydroxy-3-(4-hydroxyphenyl)-4H-1-benzopyran-4-one OR Daidzeol OR Isoaurostatin OR 7-hydroxy-3-(4-hydroxyphenyl)-4H-chromen-4-one |
| Equol | Equol OR (S)-3,4-dihydro-3-(4-hydroxyphenyl)-2H-1-Benzopyran-7-ol OR 4',7-Dihydroxyisoflavan OR 4',7-Isoflavandiol OR (3S)-3-(4-hydroxyphenyl)-3,4-dihydro-2H-1-benzopyran-7-ol |
| O-Desmethylangolensin | O-Desmethylangolensin OR 2',4'-Dihydroxy-2-(p-hydroxyphenyl)-Propiophenone OR 2,4-Dihydroxyphenyl p-hydroxyphenethyl ketone OR O-Demethylangolensin OR O-Demethylangolesin OR 1-(2,4-dihydroxyphenyl)-2-(4-hydroxyphenyl)propan-1-one |
| Glycitein | Glycitein OR 7,4'-Dihydroxy-6-methoxyisoflavone OR 7-hydroxy-3-(4-hydroxyphenyl)-6-methoxy-4H-chromen-4-one |
| Kaempferol | kaempferol OR kampferol OR kampherol OR 3,5,7-trihydroxy-2-(4-hydroxyphenyl)-4H-chromen-4-one OR 3,5,7-Trihydroxy-2-(4-hydroxyphenyl)-4H-1-benzopyran-4-one OR nimbecetin |
| Dimethylamine | Dimethylamine OR N-Methylmethanamine OR N,N-Dimethylamine OR Methanamine, N-methyl- OR Dimethylamin |
| Glutamine | (2S)-2,5-diamino-5-Oxopentanoic acid OR (2S)-2-amino-4-Carbamoylbutanoic acid OR (S)-2,5-diamino-5-Oxopentanoic acid OR Glutamic acid 5-amide OR L-(+)-Glutamine OR L-2-Aminoglutaramic acid OR L-Glutamic acid gamma-amide OR L-Glutamin OR L-Glutaminsaeure-5-amid OR Levoglutamide OR (2S)-2,5-diamino-5-Oxopentanoate OR (2S)-2-amino-4-Carbamoylbutanoate OR (S)-2,5-diamino-5-Oxopentanoate OR Glutamate 5-amide OR Glutamate amide OR L-2-Aminoglutaramate OR L-Glutamate g-amide OR L-Glutamate gamma-amide OR L-Glutamate γ-amide OR L-Glutamic acid g-amide OR L-Glutamic acid γ-amide OR 2-Aminoglutaramic acid OR Cebrogen OR gamma-Glutamine OR Glavamin OR Glumin OR L-2-Aminoglutaramidic acid OR L-Glutamic acid 5-amide OR L-Glutamid OR L-Glutamide OR Levoglutamid OR Levoglutamida OR Levoglutamidum OR Levoglutamina OR Polyglutamine OR Stimulina OR D-Glutamine |
| 3-Methylhistidine | 3-Methyl-L-histidine OR 3-Methylhistidine OR L-Histidine 3-methyl- OR 3,methylhistidine OR Tau-methylhistidine OR (S)-2-Amino-3-(1-methyl-1H-imidazol-5-yl)propanoic acid OR L-3-Methylhistidine OR H-His(3-Me)-OH OR N(pi)-Methyl-L-histidine OR N-pros-Methyl-L-histidine OR N(pai)-Methyl-L-histidine OR N(pros)-methyl-L-histidine OR (2S)-2-amino-3-(1-methyl-1H-imidazol-5-yl)propanoic acid OR 3-N-Methyl-L-histidine OR pi-Methyl-L-histidine OR 1-Methylhistidine OR Tau-Methyl-L-histidine OR N3-Methyl-L-histidine OR Histidine, 3-methyl- OR L- 1-Methyl-L-histidine OR 3-(1-Methylimidazol-5-yl)-L-alanine OR (2S)-2-amino-3-(1-methyl-1H-imidazol-5-yl)propanoate OR (2S)-2-amino-3-(3-methylimidazol-4-yl)propanoic acid |
| Trigonelline | trigonelline OR trigonellin OR gynesine OR betain nicotinate OR betaine nicotinate OR N-methylnicotinic acid OR N-methylnicotinate OR nicotinic acid N-methylbetaine |
| Pipecolic acid | pipecolic acid OR pipecolinic acid OR pipecolinate OR pipecolate OR piperidine-2-carboxylic acid OR 2-piperidinecarboxylic acid OR 2-piperidinecarboxylate OR 2-carboxypiperidine OR homoproline |
| Indolepropionate | indole propionate OR b-indolepropionate OR beta-indolepropionate OR 3-indolepropionate OR indolepropionic acid OR indole-3-propionic acid OR 1H-indole-3-propionic acid OR indole-3-propanoic acid OR 3-(3-indolyl)propionate OR beta-(3-indolyl)propionate OR beta-(3-indolyl)propionic acid OR 3-(3-indolyl)propionic acid OR 3-(1H-indol-3-yl)propionic acid OR 3-(1H-indol-3-yl)propanoic acid OR 3-(3-indolyl)propanoic acid OR 3-(1H-indol-3-yl)propanoate OR 3-(2-carboxyethyl)-1H-indole |
| *S*-Methylcysteine | L-methylcysteine OR S-methyl cysteine OR S-methyl-L-cysteine OR S-methyl-DL-cysteine OR S-11C-methyl-L-cysteine OR L-aspartic acid dimethyl ester |
| N-Acetyl-L-Ornithine | acetyl-ornithine OR *N*-acetylornithine OR N2-acetyl-L-ornithine OR N(2)-acetyl-L-ornithine OR N(delta)-acetylornithine |
